# Changes in Interictal Pretreatment and Posttreatment EEG in Childhood Absence Epilepsy

**DOI:** 10.3389/fnins.2020.00196

**Published:** 2020-03-13

**Authors:** Pawel Glaba, Miroslaw Latka, Małgorzata J. Krause, Marta Kuryło, Wojciech Jernajczyk, Wojciech Walas, Bruce J. West

**Affiliations:** ^1^Department of Biomedical Engineering, Wrocław University of Science and Technology, Wrocław, Poland; ^2^Department of Pediatric Neurology, T. Marciniak Hospital, Wrocław, Poland; ^3^Clinical Neurophysiology, Institute of Psychiatry and Neurology, Warszawa, Poland; ^4^Neonatal and Pediatric Intensive Care Unit, University Hospital in Opole, Opole, Poland; ^5^Mathematics and Information Science Directorate, Army Research Office, Durham, NC, United States

**Keywords:** childhood absence epilepsy, EEG, wavelets, biomarker, cortical excitability

## Abstract

Spike and wave discharges (SWDs) are a characteristic manifestation of childhood absence epilepsy (CAE). It has long been believed that they unpredictably emerge from otherwise almost normal interictal EEG. Herein, we demonstrate that pretreatment closed-eyes theta and beta EEG wavelet powers of CAE patients (20 girls and 10 boys, mean age 7.4 ± 1.9 years) are much higher than those of age-matched healthy controls at multiple sites of the 10–20 system. For example, at the C4 site, we observed a 100 and 63% increase in power of theta and beta rhythms, respectively. We were able to compare the baseline and posttreatment wavelet power in 16 patients. Pharmacotherapy brought about a statistically significant decrease in delta and theta wavelet power in all the channels, e.g., for C4 the reduction was equal to 45% (delta) and 63% (theta). The less pronounced attenuation of posttreatment beta waves was observed in 13 channels (36% at C4 site). The beta and theta wavelet power were positively correlated with the percentage of time in seizure (defined as the ratio of the duration of all absences which patients experienced to the duration of recording) for majority of channels. We hypothesize that the increased theta and beta powers result from cortical hyperexcitability and propensity for epileptic spike generation, respectively. We argue that the distinct features of CAE wavelet power spectrum may be used to define an EEG biomarker which could be used for diagnosis and monitoring of patients.

## Introduction

Childhood absence epilepsy (CAE) is the most common pediatric epileptic syndrome. It has a prevalence of 10–15% in childhood epilepsies and an incidence of 1.3–6 per 100,000 in children under the age of 16 years (Covanis, 2010). The disorder is most likely multifactorial, resulting from interactions between genetic and acquired factors (Gallentine and Mikati, 2012).

The ictal EEG of a typical absence seizure demonstrates rhythmic ∼3 Hz bilateral, synchronous and symmetrical spike and wave discharges (SWDs) with a median duration of approximately 10 s, which may appear several times per day, sometimes as often as dozens of times per day (Schomer and Lopes da Silva, 2018). It has long been believed that SWDs are unpredictable and emerge from otherwise almost normal interictal EEG (Lopes da Silva et al., 2003). Interictal EEG abnormalities include sparse fragments of SWDs and focal discharges as well as posterior bilateral delta activity (Sadleir et al., 2006).

The response to monotherapy (valproic acid, ethosuximide, and lamotrigine) is generally good. As many as 75–85% of treated patients are seizure free and have a normal EEG (Covanis, 2010). The rest usually respond to a combination of drugs such as valproic acid and ethosuximide. Pharmacological seizure control with acceptable side effects is achieved for slightly more than half of the children. The relationship between pretreatment EEG and response to therapy is unknown (Dlugos et al., 2013). In general, CAE carries a good prognosis. Seizures spontaneously cease with ongoing maturation.

The cortical focus theory postulates that an absence seizure originates from a focus in the somatosensory cortex which is in a specific state conducive to seizure propagation. At the beginning of the seizure, it is the cortex that drives the thalamus, but prominent generalized spike-wave discharges result from their subsequent mutual interaction (Meeren et al., 2005). Considering the principal role of the cortex in absence seizure generation, we hypothesize that in CAE, interictal EEG manifests distinct features that can be detected even in routine, rest EEG recordings.

## Materials and Methods

We retrospectively analyzed a routine, anonymized pretreatment video EEG recording (average duration 37 ± 13 min) of *n* = 30 CAE patients (20 girls and 10 boys) with a mean age of 7.4 ± 1.9 years. The analysis was approved by the ethics committee and the board of the T. Marciniak Hospital in Wrocław, Poland. CAE epilepsy syndrome was established based on of history, age at onset, clinical, and EEG findings as well as neuroimaging. In this cohort, we observed 173 seizures, 6 ± 3 per patient on average. The median seizure duration was 12 ± 4 s. On average, the ratio of the duration of all absences which patients experienced to the duration of EEG recording was 4 ± 2%. We refer to this ratio as the percentage of time in seizure (PTS). In nine cases, the first seizure occurred during hyperventilation, on average 130 ± 55 s after its onset. The control group (15 girls and 15 boys) was age matched (7.3 ± 1.9 years) to the patients. EEG was recorded using the Elmiko Digitrack system with a BRAINTRONICS B.V. ISO-1032CE amplifier. The sampling frequency was equal to 250 Hz. The 10–20 international standard was used to position 19 Ag/AgCl electrodes (impedances were below 5 kΩ). The ground electrode was placed at the patients’ forehead. The average reference electrode montage was used for time-frequency calculations. All the EEG recordings were performed between 2008 and 2018 by the same certified EEG technician.

After diagnosis, the pharmacotherapy of 16 out of 30 patients was administered by the outpatient neurology department of T. Marciniak Hospital. Valproic acid (two daily dosages of 244 ± 58 mg and 311 ± 111 mg) was used in 75% of the cases and 19% of the patients were treated with ethosuximide (two daily dosages of 250 mg). A combination of valproic acid (250 mg twice a day) and ethosuximide (150 mg twice a day) was used in 6% of the patients. The treatment response was evaluated with routine EEG with hyperventilation and photostimulation.

For each patient we extracted five closed-eyes (CE) motion artifact free pretreatment EEG segments: interictal (preceding the seizure by 226 ± 122 s), preictal A (beginning 31 s before the seizure), preictal B (beginning 16 s before the seizure), postictal A (beginning 1 s after seizure), and postictal B (beginning 16 s after the seizure). The average length of the interictal segments was: 84 ± 32 s. Due to the presence of motion artifacts and/or eye openings the length of the preictal and postictal segments slightly varied. Their average length was equal to 15 ± 2 s, 15 ± 3 s, 16 ± 1 s, and 14 ± 2 s for preictal A, preictal B, postictal A, and postictal B, respectively. The chosen length of pre- and postictal segments assured that data from all subjects could be included in analysis. For the controls, the average length of selected CE EEG segments was 55 ± 27 s. We also analyzed the most recent follow-up EEG which on average was recorded 1.5 ± 2 years after the video EEG used for diagnosis. The average length of CE segments selected for analysis from the follow-up EEG was 28 ± 15 s. There were no seizures in the follow-up EEGs.

**FIGURE 1:**
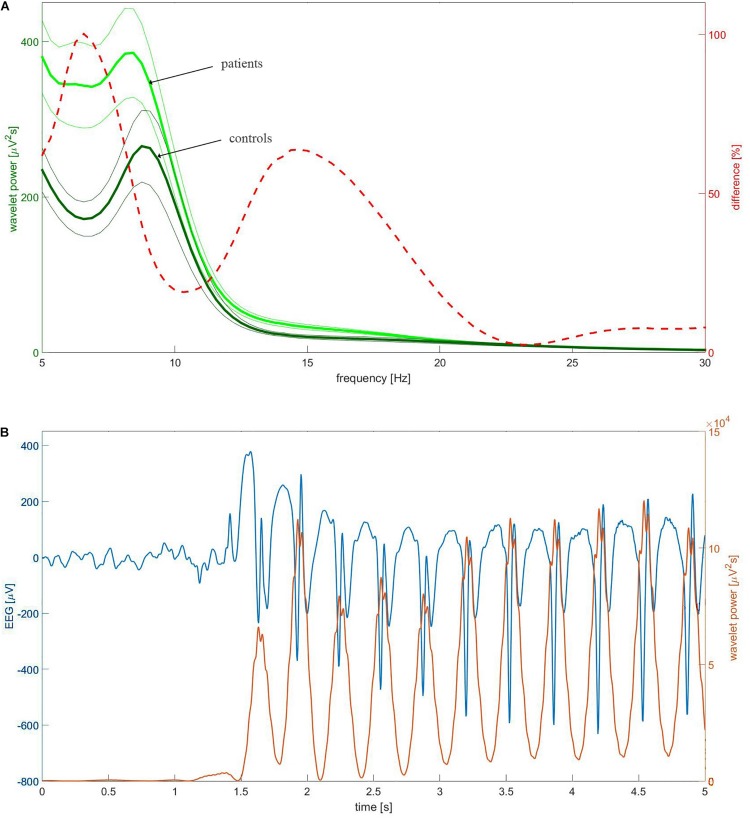
**(A)** The group-averaged wavelet power spectrum in C4 channel of patients (thick light green) and controls (thick dark green). The complex Morlet wavelet (*f_*c*_* = 1, *f_*b*_* = 1.8) was used as a mother function. The thin lines which bracket the averages represent standard error of the mean (SEM). The percentage difference between the patients and controls was drawn with the red dashed line. **(B)** Example of childhood absence epilepsy seizure. The presented section of EEG (blue curve) was extracted from EEG of a 7-year-old girl (patient BZ). The instantaneous power of the complex Morlet wavelet (*f_*c*_* = 1, *f_*b*_* = 1.8) calculated for 15 Hz was drawn with the red curve.

We calculated the continuous wavelet transform (CWT) of EEG using the complex Morlet mother function. We analyzed the statistical properties of instantaneous wavelet power *w*(*f, t*_0_) (square of the modulus of complex wavelet coefficients) and temporal average *W* (*f)* = < *w*(*f, t*_0_) > *_*t*_*_0_ over the selected EEG segment. We used the mother function with the center frequency *f_*c*_* = 1 and bandwidth *f_*b*_* = 1.8 (Latka et al., 2003). For these parameters, the transform was computed for three frequencies *f:* 6.5, 9, and 15 Hz. Figure 1A elucidates the rationale for such a choice of frequencies. The dashed red line in this figure represents the percentage difference between the C4 wavelet spectrum *W* (*f)* of patients and controls. The difference curve has two distinct local maxima at 6.5 and 15 Hz. These frequencies were used to analyze theta and beta rhythms, respectively. The spectra of patients and controls have the maxima around 9 Hz and consequently such frequency was used for analysis of alpha waves. Figure 1B shows that the chosen wavelet calculated for 15 Hz may be used for detection of epileptic spikes. In other words, we focus on this part of the beta band which may be involved in the genesis of epileptic spikes. The spectacular increase of beta wavelet power in the vicinity of the epileptic spikes in Figure 1B can be employed in the simple but highly effective SWD detector which we describe elsewhere. The wavelet with *f_*c*_* = 1 and bandwidth *f_*b*_* = 1.5 calculated for frequency 3 Hz was used to quantify delta waves.

We used the Shapiro–Wilk test to determine whether the analyzed data were normally distributed. We compared the pretreatment and posttreatment values of *W* (*f)* as well as the values of the controls with the Kruskal–Wallis test (with Tukey’s *post-hoc* comparisons). The same test was used to analyze the changes in wavelet power in preictal and postictal segments. The patients’ pretreatment wavelet power averaged over all channels was compared with that of controls using also a non-parametrical statistical testing (Maris and Oostenveld, 2007). The reported *p*-values for this test correspond to 1 × 10^5^ random draws. The area under the receiver operating characteristic curve (AUC) was used to quantify differences in *W*(*f)* between patients and controls. AUC was also calculated for pretreatment and posttreatment values of *W*. The values of AUC, sensitivity and specificity were reported for selected channels. The correlation between the values of *W*(*f)* for all four EEG bands and the percentage of time in seizure was examined for all 19 EEG channels using Pearson’s correlation coefficient. The significance threshold for all the statistical tests was set to 0.05. The wavelet and statistical analyses were done with MATLAB R2015A.

We employed classifiers implemented in Weka software (version 3.8)^1^ to discriminate between patients and controls using theta and beta wavelet power *W*(*f)*. Out of 38 such attributes, we used only these with non-zero gain ratio (entropic measure). Machine learning was performed with 10-fold cross-validation.

## Results

**FIGURE 2:**
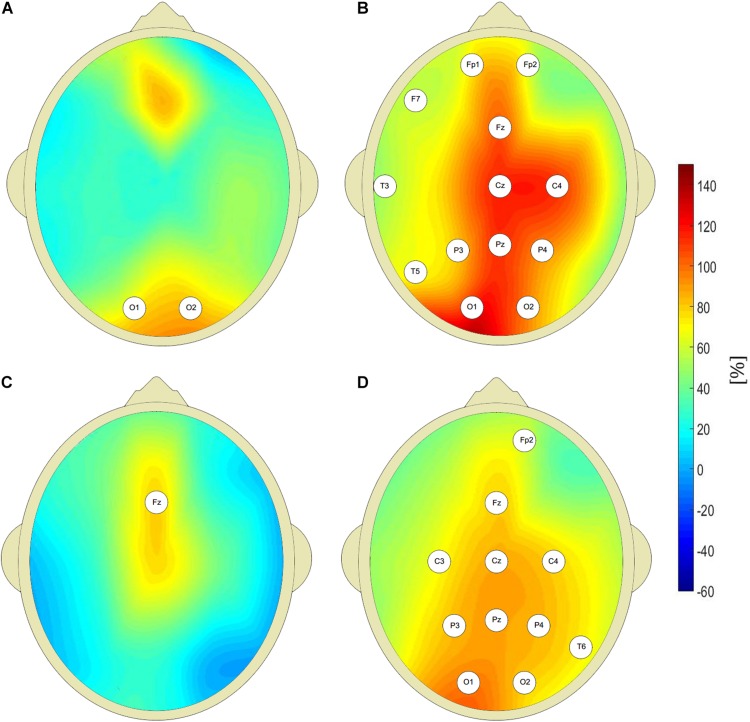
Percentage differences between wavelet power of closed-eyes interictal pretreatment EEG of CAE patients and the controls (with respect to controls) for: delta **(A)**, theta **(B)**, alpha **(C)**, and beta **(D)** rhythms. The EEG channel labels indicate sites for which the differences were statistically significant.

Figure 2 shows that the pretreatment interictal closed-eyes theta and beta EEG wavelet powers of CAE patients are much higher than those of age-matched controls at multiple sites of the 10–20 system. For the theta band, we observed a 100% increase in patients’ wavelet power *W* at C4 site (343 ± 296 μV^2^s vs. 172 ± 118 μV^2^s, *p* = 9 × 10*^–^*^3^, AUC = 0.87, sensitivity = 0.87, specificity = 0.63). For the beta band, the power in the patients was 63% greater than in the controls (32 ± 18 μV^2^s vs. 20 ± 13 μV^2^s, *p* = 4 × 10*^–^*^3^, AUC = 0.77, sensitivity = 0.77, specificity = 0.73). With the exception of channel Fz, there was no statistically significant difference between the pretreatment patients and the controls for alpha rhythm. For the delta band, the increased pretreatment wavelet power was observed only at the occipital channels.

The value of *W(f)* averaged over all 19 EEG channels was higher for pretreatment patients both for theta and beta bands. The corresponding *p*-values for Kruskall-Wallis test were equal to 4 × 10^–3^ and 1 × 10^–3^. For non-parametric testing, *p* = 8 × 10^–4^ and *p* = 1 × 10^–4^ for theta and beta rhythms, respectively. Kruskall-Wallis test did not detect differences in alpha and delta power between untreated patients and controls. Non-parametric testing showed the differences in the delta band with *p* = 2 × 10^–2^.

**FIGURE 3:**
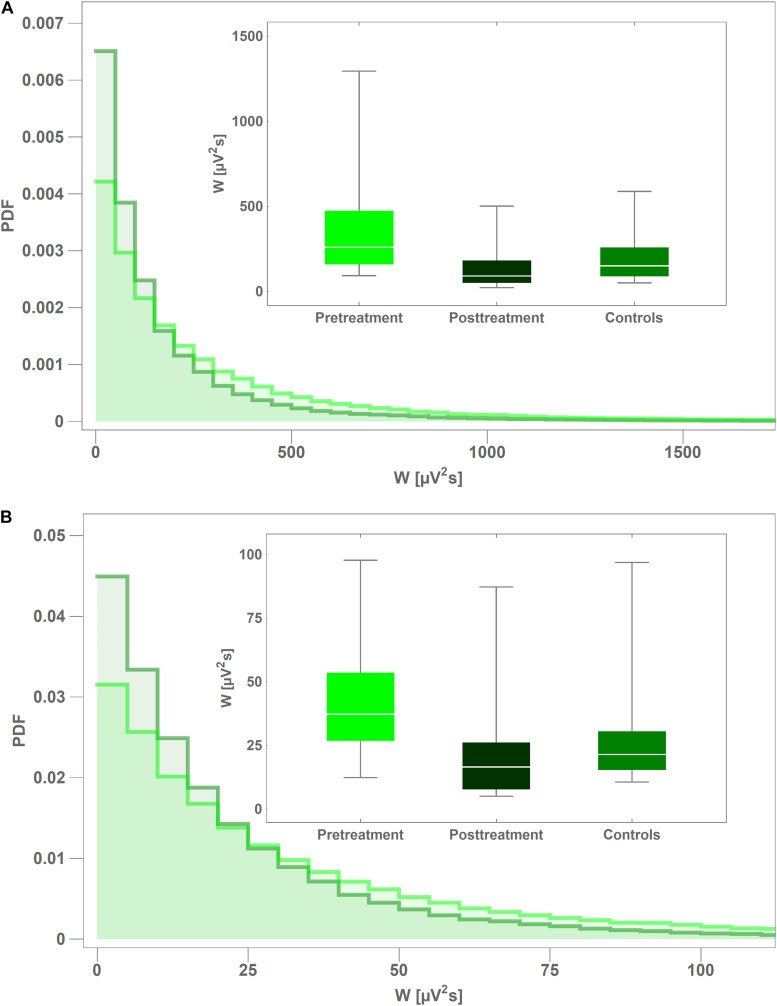
Comparison of probability density function (PDF) of instantaneous wavelet power of interictal pretreatment EEG of CAE patients (light green) and the controls (dark green) for: theta **(A)** and beta **(B)** rhythms. PDFs were calculated from the closed-eyes segments extracted from C4 channel. The insets in both subplots show the boxplots of time-averaged values of the pretreatment and posttreatment wavelet power of as well as that of the controls.

In Figure 3 we present the probability density function (PDF) of instantaneous interictal theta and beta wavelet power *w(f,t_0_)* in channel C4 for the controls (light green) and the pretreatment patients (dark green). For both cohorts, we aggregated the values of *w(f,t_0_)* from all their members. It is apparent that the tail of the PDF is much longer for the pretreatment cohort. The insets in both subplots show the boxplots of time-averaged values of the pretreatment and posttreatment wavelet power *W* as well as that of the controls. It is apparent from Figure 3A that pharmacotherapy suppressed the elevated pretreatment theta power that dropped 63% from 343 ± 296 μV^2^s to 127 ± 123 μV^2^s with *post-hoc p* = 2 × 10*^–^*^4^ (AUC = 0.87, sensitivity = 0.75, specificity = 0.87). The patients’ posttreatment C4 theta power was not statistically different from that of the controls (*p* = 0.26). Figure 3B shows that the influence of treatment on C4 beta power was weaker. In that case the power was reduced 36% from 32 ± 18 μV^2^s to 21 ± 22 μV^2^s with *post-hoc p* = 3 × 10*^–^*^3^ (AUC = 0.78, sensitivity = 0.81, specificity = 0.70).

For the alpha band, there were no statistically significant differences between the wavelet power in interictal segments and those in preictal and postictal ones. The *W* of theta and beta rhythms was statistically greater than the interictal value only in postical A segments (channels Fp1, Fp2, F7, F8, and Fp1, F7, respectively). Such increases in theta wavelet power were not observed in postictal B segments. For preictal B and postictal A segments (adjacent to the absence seizure) the delta wavelet power was statistically greater than the interictal values in all 19 EEG channels. For example, at the C4 site, patients’ wavelet power in preictal B segment was 367% greater than in the controls (1,259 ± 1,973 μV^2^s vs. 269 ± 136 μV^2^s, *p* = 3 × 10*^–^*^5^, AUC = 0.82, sensitivity = 0.76, specificity = 0.83) and 212% greater than the average value in the interictal segments 403 ± 276 μV^2^s (*p* = 2 × 10*^–^*^2^, AUC = 0.82, sensitivity = 0.69, specificity = 0.73). Postictal A wavelet power *W* at C4 for the delta band was 651% greater than in the controls (2,020 ± 3,837 μV^2^s vs. 269 ± 136 μV^2^s, *p* = 1 × 10*^–^*^5^, AUC = 0.83, sensitivity = 0.76, specificity = 0.83) and 402% greater than that for interictal segments (*p* = 1 × 10*^–^*^2^, AUC = 0.74, sensitivity = 0.69, specificity = 0.77). There were no statistically significant differences in the interictal and postictal B delta wavelet power. Thus, the increase in postictal theta and delta wavelet power subsides within 30 s after the absence seizure.

Both for the theta and beta bands, the interictal wavelet power *W* was positively correlated with the percentage of time in seizure PTS in majority of channels. For theta waves, there were 17 such channels and the average value of Pearson’s correlation coefficient *r* was equal to 0.51 ± 0.11 (range 0.26–0.66, maximum at T3 and minimum at P4). For beta rhythm, we found 16 channels where correlations were significant (average value *r* = 0.51, range 0.19–0.66, maximum at P3 and minimum at O2). No statistically significant relationship between *W* and PTS was observed for alpha waves.

**FIGURE 4:**
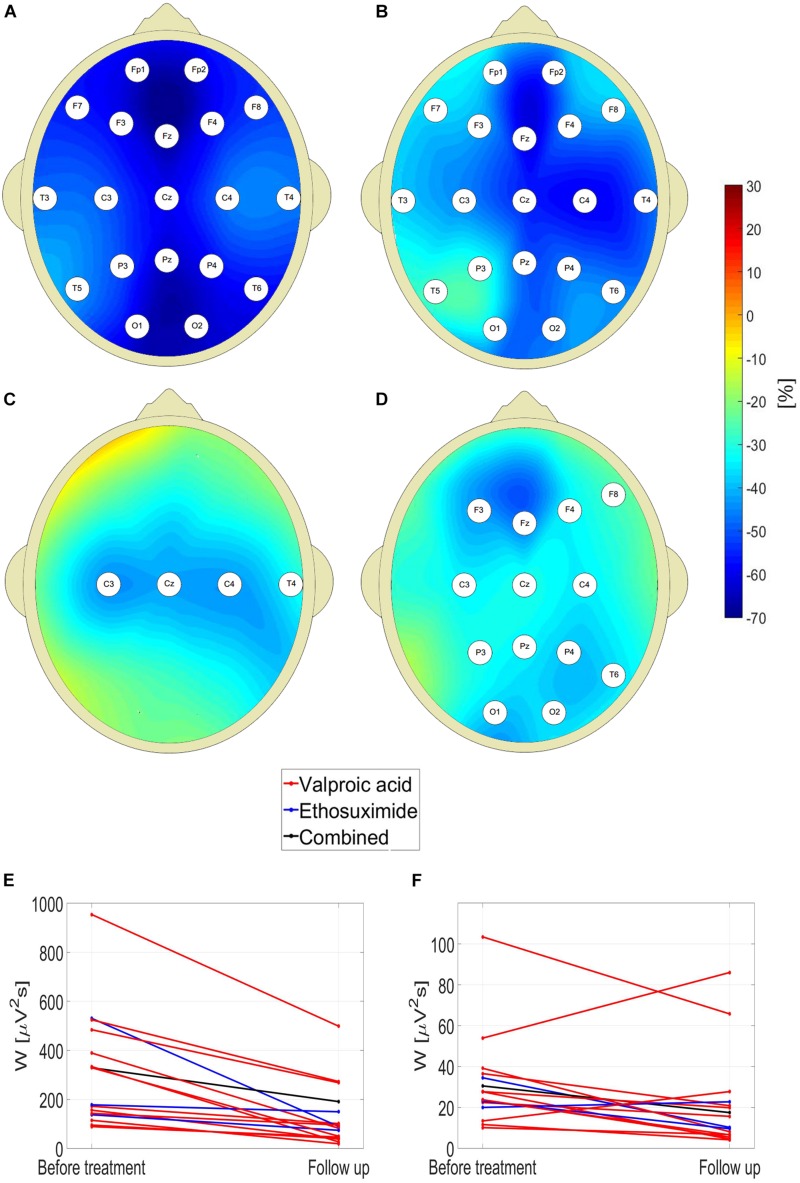
Percentage change in posttreatment closed-eyes wavelet power of CAE patients with respect to the baseline for: delta **(A)**, theta **(B)**, alpha **(C)**, and beta **(D)** rhythms. The EEG channel labels indicate sites for which the differences were statistically significant. Effect of treatment on **(E)** theta and **(F)** beta wavelet power *W* in C4 channel.

One can see in Figure 4 that pharmacotherapy primarily attenuates the low-frequency part of EEG spectrum. For all the channels, the values of interictal posttreatment delta and theta power are smaller than the pretreatment ones. In particular, for the delta band, *W* for C4 channel fell 45% from 394 ± 280 μV^2^s to 218 ± 328 μV^2^s (*p* = 7 × 10*^–^*^3^, AUC = 0.90, sensitivity = 0.75, specificity = 0.84). The less pronounced decrease in posttreatment beta power was observed in 13 channels. The fact that there is no increase of posttreatment alpha wavelet power implies that the attenuation of theta rhythm does not result from EEG maturation but is caused by treatment.

For channel C4, the reduction of *W* was observed in 100 and 81% patients for the theta and beta band, respectively. The data presented in Figures 4E,F are representative of the downward trend in the wavelet power during pharmacotherapy. For theta rhythm, a reduction was observed in all patients for channel C4, and all but one in channels: F7, T5, T6, P3, P4, and Pz. Upon averaging of all channels, both for theta and beta waves the reduction of *W* was observed in 94% cases.

Out of 38 potential attributes, only 13 had non-zero gain ratio. For theta rhythm: C4 (0.28), Cz (0.28), P3 (0.24), O1 (0.22), Fz (0.21), Pz(0.21), C3 (0.20), For beta waves: Pz (0.29), P3(0.28), P4 (0.28), O1 (0.26), O2 (0.21), C4 (0.19). The Bayesian network classifier (with default parameters) turned out to be the most balanced. It achieved accuracy of 78%, specificity 79%, and sensitivity 77%. The classification was based on only six channels: C4, Cz, Fz for theta band and Pz, P3, O1 for beta band.

## Discussion

GAERS and the WAG/Rij rodent models have paved the way for the understanding of pathophysiology of human CAE seizures (Akman et al., 2010). Lüttjohann and Van Luijtelaar reviewed animal studies and the translation of research results from rodent models to humans (Lüttjohann and Van Luijtelaar, 2015). According to the cortical focus theory of Meeren et al. (2005) the genesis of absence seizure starts with a single spike which rapidly spreads over the hyperexcited cortex. The formation of prominent SWDs is possible only due to interaction with the thalamus which acts as a resonant circuit. The rapid generalization of spike-wave activity over the cortex is due to short-range intracortical fibers and to a subpopulation of cells that have long-range association fibers. These fibers run under the cortex in the white matter, making extensive connections with other cortical areas.

In rats, SWDs emerge from the cortical focus which is located either within the perioral somatosensory cortex (WAG/Rij) or the secondary somatosensory cortex (GAERS) and subsequently propagate via the corticothalamocortical loop. Polack et al. demonstrated that blockade of action potential discharge and synaptic activities in facial somatosensory cortical neurons (by topical application of tetrodotoxin) prevents the formation of SWDs (Polack et al., 2009). In contrast, pharmacological inhibition of a remote motor cortical region does not suppress ictal activities. Westmijse et al. discovered, using a beamforming source localization technique, that in humans the sources of spikes from a train of SWDs were at the frontal lateral, central and medial parietal cortices (Westmijse et al., 2009). The involvement of the thalamus in the generation of SWDs was demonstrated in combined EEG-fMRI (Granert et al., 2008; Moeller et al., 2010) and MEG (Tenney et al., 2013) studies. The differences between rat and human data are in the frequencies of SWDs (6–11 Hz vs. 2.5–4 Hz) and the location of the early local cortical activity which is quite variable in humans. The location may even change during a seizure (Westmijse et al., 2009) and is predominantly, but not exclusively, located in the frontal-central/parietal areas. The low variability of the position of cortical focus in the rats can easily be explained by the fact that both epileptic strains are fully inbred, and the animals are homozygous.

Herein, we found that the pretreatment closed-eyes theta wavelet power of CAE patients was much higher than that of age-matched controls at multiple sites of the 10–20 system. Moreover, the theta wavelet power was positively correlated with the percentage of time in seizure. This result should not come as a surprise. In the late 1960s Doose et al. argued that strong rhythmic theta activity was an age-dependent electroencephalographic expression of a genetic disposition to convulsions, see a review paper (Doose and Baier, 1988) for a complete list of references. This hypothesis was noted, along with the opposing view, by the authors of the classic text on encephalography (Schomer and Lopes da Silva, 2018). To the best of our knowledge, initial observations have not been followed up on using quantitative EEG analysis (Doose et al., 2003). The more recent research provided new evidence for the role of theta rhythm in epilepsy. Clemens et al. (2012) using a Low Resolution Electromagnetic Tomography (LORETA) source localization found increased theta activity in the basal prefrontal and medial temporal limbic areas of CAE patients. Dömötör et al. (2017) observed reduced current source density in 0.5–8 Hz frequency range in CAE patients who responded to pharmacotherapy. Douw et al. (2010) in their MEG studies found that theta band brain connectivity and network topology is altered in epilepsy which developed in brain tumor patients. Milikovsky et al. (2017) discussed the properties of theta dynamics in the animal model of postinjury epilepsy. Sitnikova and van Luijtelaar (2009) found that the increased delta and theta power preceded SWDs in WAG/Rij rats.

We interpret the results presented herein as evidence in support of the following postulates. We hypothesize that the increased theta rhythm power in CAE patients is a manifestation of cortical hyperexcitability (Vestal and Blumenfeld, 2010). Since strong theta rhythm may be found in up to 15% of healthy children (Doose and Baier, 1988), cortical hyperexcitability may be a necessary but not a sufficient prerequisite for CAE. The increased beta power may reflect propensity for epileptic spikes generation – spikes that trigger absence seizures. This interpretation is corroborated by the recent work of Sorokin et al. (2016) who found that in rats absence seizure susceptibility correlates with pre-ictal beta oscillation. It is worth pointing out that as we detune the parameters of the analyzing wavelet away from the values optimal for spike detection, the differences in beta power between the pretreatment patients and controls disappear.

As with all epileptic syndromes, once the diagnosis of CAE is confirmed, only the absence or recurrence of seizures provides an indication as to whether anti-epileptic drugs (AEDs) have had any effect. We argue that the distinct features of CAE wavelet power spectrum may be used to define an EEG biomarker which could be used for diagnosis and long-term monitoring of patients. The most apparent applications of such monitoring would be in the assessment of the efficacy of pharmacotherapy and its duration.

Over the last three decades, transcranial magnetic stimulation (TMS) has become a principal tool for accessing cortical excitability associated with epilepsy (Bauer et al., 2014). However, there are essentially no TMS studies of drug-naive patients with CAE (Kessler, 2016). The barriers to the inclusion of children from such studies stem not only from the very nature of this experimental technique, e.g., its duration or immobilization of head during measurement, but also from a few fundamental reasons. For example, it is not clear whether the motor cortex physiology is a reliable marker of seizure susceptibility in children with less mature brain networks. Moreover, abnormalities in TMS markers of cortical excitability are not specific to epilepsy and may observed, among others, in ADHD, a common comorbid condition in children with epilepsy (Reilly et al., 2017). Future research should verify whether measurement of theta band power in CAE patients could be used in lieu of TMS. In particular, the previous TMS studies (Wright et al., 2006; Badawy et al., 2009) revealed that increased excitability preceded epileptic seizures. Thus, the question arises as to whether temporal changes in cortical excitability occur in CAE.

To use theta and beta wavelet power as biomarkers of CAE it needs to be proved that the observed changes between patient and controls are reproducible (which is important considering inherent variability of human EEG) and are not affected by the developmental changes of EEG which occur about the age of puberty. The main limitation of the study comes from its retrospective character. We analyzed the routine video EEGs that were used for diagnosis. We were able to demonstrate that the differences between patients and controls persisted throughout the EEG recordings. In particular, there were no differences in theta and beta wavelet power between the interictal segment (preceding the first observed seizure) and the preictal A, and postictal B segments. The data showed that the changes in wavelet power associated with absence seizure subsided within 30 s after its end. As we already mentioned, cortical excitability is known to vary with time. There is no doubt that future long-term EEG monitoring must verify our findings. The mean age of subjects was 7.4 ± 1.9 years. The follow-up EEGs were recorded on average one and a half year later. Puberty usually occurs in girls between the ages of 10 and 14, while in boys it generally occurs later. Thus, it is unlikely that the observed reduction of delta and theta power was merely effect of maturation and not brought about by pharmacotherapy. Moreover, there were no increases in alpha wavelet power in pretreatment and posttreatment EEGs which argues against the maturation effect.

Even though the analyzed pretreatment EEG segments were short, we were able to construct a balanced classifier which differentiated patients and controls with good accuracy using only several attributes. The assessment of applicability of such classifier in absence seizure diagnostics requires further studies with much larger datasets.

Pharmacological seizure control with acceptable side effects is achieved for slightly more than half of children with CAE. Herein we found that pharmacotherapy most effectively suppresses low-frequency EEG oscillations. Quantitative EEG analysis offers a unique opportunity to design a treatment which would more selectively attenuate the pathological theta and/or beta rhythms. The availability of low-cost, Internet connected personal EEG devices paves the way for home monitoring of patients which would facilitate drug titration and termination of pharmacotherapy.

## Data Availability Statement

The raw data supporting the conclusions of this article will be made available by the authors, without undue reservation, to any qualified researcher.

## Ethics Statement

The studies involving human participants were reviewed and approved by the ethics committee of T. Marciniak Hospital in Wrocław, Poland. Written informed consent from the participants’ legal guardian/next of kin was not required to participate in this study in accordance with the national legislation and the institutional requirements.

## Author Contributions

MJK, ML, and PG: conceptualization. PG, MK, MJK, and ML: investigation and original draft preparation. BW, WW, and WJ: review and editing. All authors contributed to methodology and formal analysis.

## Conflict of Interest

The authors declare that the research was conducted in the absence of any commercial or financial relationships that could be construed as a potential conflict of interest.

## Footnotes

1 https://www.cs.waikato.ac.nz/ml/weka/
